# Outcome prediction based on microarray analysis: a critical perspective on methods

**DOI:** 10.1186/1471-2105-10-53

**Published:** 2009-02-07

**Authors:** Michalis Zervakis, Michalis E Blazadonakis, Georgia Tsiliki, Vasiliki Danilatou, Manolis Tsiknakis, Dimitris Kafetzopoulos

**Affiliations:** 1 Technical University of Crete, Department of Electronic and Computer Engineering, University Campus, Chania Crete 73100, Greece; 2 Post-Genomic Laboratory, Institute of Molecular Biology and Biotechnology, FORTH, Vassilika Vouton, 71110, Heraklion, Crete, Greece; 3 Biomedical Informatics Laboratory, Institute of Computer Science, FORTH, Vassilika Vouton, 71110, Heraklion, Crete, Greece

## Abstract

**Background:**

Information extraction from microarrays has not yet been widely used in diagnostic or prognostic decision-support systems, due to the diversity of results produced by the available techniques, their instability on different data sets and the inability to relate statistical significance with biological relevance. Thus, there is an urgent need to address the statistical framework of microarray analysis and identify its drawbacks and limitations, which will enable us to thoroughly compare methodologies under the same experimental set-up and associate results with confidence intervals meaningful to clinicians. In this study we consider gene-selection algorithms with the aim to reveal inefficiencies in performance evaluation and address aspects that can reduce uncertainty in algorithmic validation.

**Results:**

A computational study is performed related to the performance of several gene selection methodologies on publicly available microarray data. Three basic types of experimental scenarios are evaluated, i.e. the independent test-set and the 10-fold cross-validation (CV) using maximum and average performance measures. Feature selection methods behave differently under different validation strategies. The performance results from CV do not mach well those from the independent test-set, except for the support vector machines (SVM) and the least squares SVM methods. However, these wrapper methods achieve variable (often low) performance, whereas the hybrid methods attain consistently higher accuracies. The use of an independent test-set within CV is important for the evaluation of the predictive power of algorithms. The optimal size of the selected gene-set also appears to be dependent on the evaluation scheme. The consistency of selected genes over variation of the training-set is another aspect important in reducing uncertainty in the evaluation of the derived gene signature. In all cases the presence of outlier samples can seriously affect algorithmic performance.

**Conclusion:**

Multiple parameters can influence the selection of a gene-signature and its predictive power, thus possible biases in validation methods must always be accounted for. This paper illustrates that independent test-set evaluation reduces the bias of CV, and case-specific measures reveal stability characteristics of the gene-signature over changes of the training set. Moreover, frequency measures on gene selection address the algorithmic consistency in selecting the same gene signature under different training conditions. These issues contribute to the development of an objective evaluation framework and aid the derivation of statistically consistent gene signatures that could eventually be correlated with biological relevance. The benefits of the proposed framework are supported by the evaluation results and methodological comparisons performed for several gene-selection algorithms on three publicly available datasets.

## Background

Modern biological and biomedical research has been challenged by the relatively new high-throughput methods of genomic, proteomic and metabolomic analysis [1], such as DNA microarrays that allow the simultaneous measurement of the expression of every gene in a cellular genome. Two of the fundamental tasks in this area are the identification of differentially expressed genes between two or more conditions and the selection of a subset of features (genes) with the best predictive accuracy for a certain classifier [2]. Various statistical methods for the analysis of microarray data exist, however data derived from these methods are complex, hard to reproduce and require expedient statistical analysis to minimize errors and avoid bias.

Despite the plethora of methods that have been developed for information extraction from microarrays, such information has not yet been widely used in diagnostic or prognostic decision-support systems [3]. This is partly due to the inconsistency of the derived results [4] and the different properties of various data sets [5-8]. For example, to correctly distinguish the two types of leukemia, Golub et al. [9] used a filter method and succeeded to derive a 50-gene signature, whereas Guyon et al. [10] applied a wrapper method in combination with SVM on the same data set and succeeded to derive an 8-gene signature.

Many studies have addressed the different issues involved in data analysis from different microarray studies [11-14]. Here we briefly mention some of those, namely the experimental platform used, the design of the study, the normalization techniques employed or even the different properties of the data distribution [5,10,15]. Also, from the point of the statistical analysis of the data, different study outcomes can be due to the different algorithms used, the improper use of validation techniques and the optimization techniques for the prediction model.

Additionally, various statistical issues can potentially affect the results of a study. For example, the bootstrapping strategy for the generation of random folds for training and testing is an issue of particular importance for performance comparison of gene selection approaches. Moreover, cross-validation (CV) can induce a certain bias due to mixing of training and testing samples [16]. Leave-one-out CV derives over-optimistic estimates, while three-fold CV split may lead to a small number of training samples and hence the possibility of overtraining [17]. The low performance of hard-margin SVM in [5] as compared to [10] on the independent test-sets may be partially attributed to such overtraining. Stratified re-sampling of data may be used to maximize the power of comparison among methods [18], but it can still modify the prior data distributions leading to changes in performance estimates. Similarly, random data splits often induce bias in case-specific considerations because they randomly exclude samples from the training or the testing process [19,20]. Thus, there is a need to consider issues of influence in algorithmic performance and associate results with confidence intervals meaningful to clinicians, in order to thoroughly compare methodologies under the same experimental and methodological set-up [16,20-22].

### Objectives of the study

Through our study we aim to reveal shortcomings in the evaluation of gene selection approaches and address methodological issues that may lead to more objective evaluation schemes. Furthermore, we consider sample-specific effects on the performance and stability of prediction algorithms. For that purpose, we have selected three well known publicly available data sets, namely the Van't Veer et al. [23], the Golub et al. [9] and the Alon et al. [15] data sets. Further details about the data sets used are given in the following section.

Our ultimate objective, besides presenting and highlighting the advantages and disadvantages of the tested methodologies, is to explore fundamental characteristics in the analysis of microarray data sets and identify methodological aspects that may influence the evaluation of algorithms. As a performance validation scheme we consider ten-fold CV repeated ten times, i.e. 100 iterations in total. It is worth recalling that CV with multiple gene-sets or different splits of data sets always presents some bias threats [16,24]. In order to relieve such biases we consider testing on an independent test-set at each step of the recursive validation process. Finally, in order to avoid bias due to the classification model, we estimate its parameters through optimization on the independent test-set.

## Results

### Data sets

Three well known and publicly available data sets first published at, [9,15] and [23] were considered. For the first data set genes were hybridized using two-color arrays, whereas for the other two one-color arrays were used. The lists of genes, after platform-specific handling of the data, were used as suggested by the corresponding authors. Thus, platform specific effects were not considered.

The breast cancer (BC) data set [23] contains 24,481 genes and 78 samples on the training set, 34 of which are characterized positive and 44 negative accordingly to the presence or not of a relapse within a period of five years. 293 genes expressing missing information for all 78 patients were removed and the remaining 13,604 missing values were substituted using Expectation Maximization (EM) imputation [25]. This set is used either as a training set or for the design of the CV trials and the specification of the bootstrap training and testing subsets. The independent test-set consists of 19 samples, 7 negative and 12 positive.

The leukemia data set in [9] consists of 38 bone marrow samples obtained from acute leukemia patients at the time of diagnosis. The data set is divided into 27 samples of acute lymphoblastic leukemia (ALL) and 11 samples of acute myeloid leukemia (AML). Data was analyzed using an Affymetrix arrays which contained 7,129 human genes. The independent test set consists of 34 samples (20 ALL and 14 AML).

The colon cancer (CC) data set in [15] consists of 40 tumour and 22 normal colon tissue samples analyzed with Affymetrix oligonucleotide array complementary to more than 6,500 human genes. 2,000 genes were considered as suggested by [15] based on their minimal intensity across samples. To create the training set, 28 tumour and 16 normal samples were randomly selected, while the remaining samples constituted the independent set.

### Data set analysis

**Figure 1:**
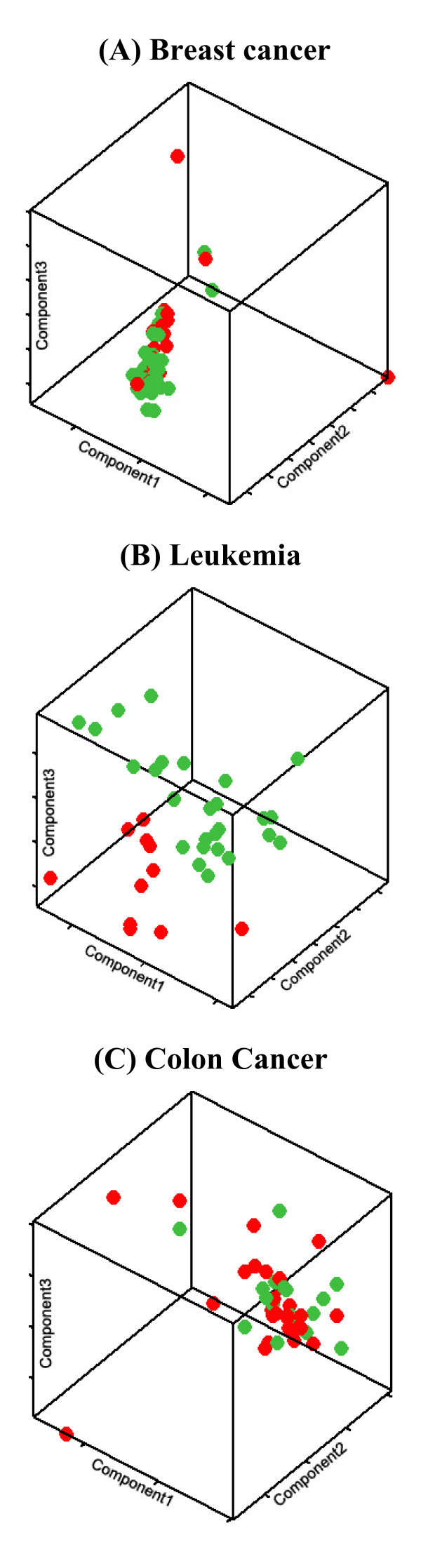
**Principal component analysis**. Three-Dimensional Principal Component Analysis (PCA) on the three examined data sets reveals that breast cancer (A) demonstrates the largest degree of overlap between the two classes, with colon cancer (C) coming next and leukemia (B) being the easiest one in locating a decision boundary.

To highlight differences between the three data sets, a three-dimensional principal component analysis (PCA) depicted in Figure 1 was performed, which could assist us in estimating a decision boundary. Such an analysis revealed that the BC data set perhaps demonstrates the highest degree of overlap between the two classes. CC data set appears to be more separable than BC, while the leukemia data set appears to be most easily distinguishable.

### Design of experimental scenarios

Three basic types of experimental scenarios are conducted in order to estimate the performance of tested methodologies:

• In the *independent test-set scenario *the algorithms are trained using the complete training set together with backward feature elimination process. Thus, a varying number of genes are recursively eliminated until 100 surviving genes are left and subsequently one gene is eliminated from this point onwards [26]. At each stage of the elimination process, the classifier is trained on the training-set using the selected set of genes, while its performance with respect to accuracy is measured on the independent test-set using the same set of genes. Finally, the minimal set of genes which achieves the maximum classification accuracy and classifies perfectly the training-set is the final set of marker genes with the corresponding maximum accuracy performance on the independent test-set. Here, we also measure the Q-statistic score [27] to investigate the level of gene differentiation among the classes of interest and gene correlation with the disease outcome. This scenario is essentially used for the estimation of algorithmic parameters and of the size of gene signature with high accuracy and good generalization on the independent test-set.

• *The maximum performance on a 10-fold CV scenario *is used to access optimal algorithmic performance under CV, similar to the GEMS approach [28,29]. The initial set of training samples is randomly partitioned into ten groups. Each group, denoted by fold for the rest of this paper, is iteratively used for testing, whereas the remaining folds are used for the training of the algorithms. Thus, 90% of the set is used for training purposes, while the remaining 10% is used as the test-set. This CV process is repeated ten times resulting in total to 100 test iterations, i.e. *10(folds) *× *10(splits per fold)*. Each iteration proceeds independently with gene selection according to the previous cut-off strategy. The maximum performance for each run on the testing subset is reported along with the corresponding number of surviving genes. Finally, we report the grand average of maximum accuracies along with the corresponding number of gene-sets and confidence intervals (CIs) on classification accuracy.

• *The average performance on a 10-fold CV scenario *operates with fixed parameters of the tested algorithms and terminates at a fixed number of selected genes determined by the independent test-set scenario. Feature selection is performed within each run of the CV process in order to avoid overestimation of the prediction accuracy [30,31]. Testing is performed on the test folds of CV, but also on the common independent test-set. In this scenario, average accuracy measures are estimated in an attempt to reduce random correlation effects and increase the confidence that the test measurements reflect the true predictive ability of each method. CIs on classification accuracy are used, where appropriate, as a stability measure revealing the variation in the performance of the tested methodologies. Furthermore, we report the number of commonly selected genes as an indicator of the stability of the tested methodologies over different training-sets. The frequency of gene appearance in classification rules has also been used by [19,20] in order to control the rate of genes selected at random.

Except from measures on the overall population used in CV, we also resort to case-specific considerations to increase the clinical relevance of algorithmic accuracy estimates. The accuracy, usually measured over many iterations, cannot evaluate the efficiency of the algorithm to correctly classify individual cases. Thus, high accuracy may be due to correct classification of different cases in each run, providing an overall high score for this measure even though single cases may not be correctly classified in most runs of the algorithm. However, when considering clinical evaluation of a new case, it is important to have high confidence in the correct categorization of the individual subject based on the training examples. For that reason it is essential to consider per subject accuracy measures, which assist in increasing the efficiency of the algorithm to correctly identify new cases with similar attributes, as those involved in the training set. Through such measures, we can also consider the stability and generalization ability of each algorithm on the basis of per subject success or failure, as well as account for the influence of entire-sample measurement errors on the estimation of the prediction power of each method. All those measures are presented along with appropriate CIs derived from the CV process.

For the effective evaluation of measures a performance profile of each algorithm is computed, i.e. a table with columns reflecting all subjects and rows indicating the CV runs. For each run (row), the table captures a binary value for each case (column) if that subject is in the test-set of the run. This value indicates the prediction success for that specific subject on the specific run. In this form, the average of per subject accuracies over all runs reflects the per subject accuracy of the algorithm, whereas the average of per run accuracies over all testing cases reflects its per run accuracy. The notation of such accuracy measures is specified in subsequent sections.

Three classes of methods have been chosen for selecting the number of genes with best performance in sample classification, namely filter, wrapper and hybrid methods. Filter and wrapper methods have been extensively used for gene selection, whereas hybrid methods have also shown considerable success by combining the advantages of the previous classes [26]. Filter methods reveal the discriminatory ability of each gene by employing various criteria such as the Fisher's criterion which is also used in the present study. Wrapper methods traditionally employ SVM classifiers in a recursive feature elimination (RFE) mode to reveal the prediction ability of groups of genes, but other (rather simple) predictors have also been tested. In our study wrapper methods were employed via five algorithms, and in particular the RFE based on Linear Neuron Weights using Gradient Descent (RFE-LNW-GD), the RFE based on Support Vector Machines (RFE-SVM), the RFE based on Least Square Support Vector Machines (RFE-LSSVM), the RFE based on Ridge Regression (RFE-RR) and the RFE based on Fisher's Linear Discriminant Analysis (RFE-FLDA). Hybrid methods are examined via RFE based on Linear Neuron Weights (RFE-LNW1, RFE-LNW2), and RFE based on Fisher's ratio of Support Vectors using a 7 Degree polynomial Kernel (RFE-FSVs-7DK). More details can be found in Methods section.

### Maximum Performance Results

#### Independent Test-set

The maximum performance results of filter, wrapper and integrated schemes on the independent test-set are presented in Tables 1, 2 and 3. Accuracy measures, as defined in Methods section, are presented along with sensitivity and specificity measures, reflecting the ability to correctly classify good and bad prognosis, respectively. The Q-statistic measure and the number of selected genes for achieving maximum performance on the independent test-set are also assessed.

**Table 1:** Maximum Performance results on the independent test-set under the first testing scenario, Van't Veer et al. [23] (breast cancer) data set.

	**Filter Method**	**Wrapper Methods**					**Hybrid Methods**		
Criterion	Fisher's Ratio	RFE-LNW-GD	RFE-SVM	RFE-LSSVM	RFE-RR	RFE-FLDA	RFE-LNW1	RFE-LNW2	RFE-FSVs-7DK

Accuracy	0.74	0.84	0.79	0.79	0.89	0.89	0.89	0.74	0.95
Sensitivity, Specificity	0.75, 0.71	0.83, 0.86	0.75, 0.86	0.83, 0.71	1.0, 0.71	0.92, 0.86	0.92, 0.86	0.75, 0.71	0.92, 1.00

Q-Statistic quality measure	135.78	81.21	52.12	12.91	82.60	42.58	99.05	131.78	112.43

Number of genes selected	61	22	32	45	7	28	44	64	73

**Table 2:** Maximum Performance results on the independent test-set under the first testing scenario Golub et al. [9] (leukemia) data set.

	**Filter Method**	**Wrapper Methods**					**Hybrid Methods**		
Criterion	Fisher's Ratio	RFE-LNW-GD	RFE-SVM	RFE-LSSVM	RFE-RR	RFE-FLDA	RFE-LNW1	RFE-LNW2	RFE-FSVs-7DK

Accuracy	0.91	0.94	0.94	0.85	0.82	0.82	0.94	0.94	0.94
Sensitivity, Specificity	0.79, 1.00	0.86, 1.00	0.86, 1.00	0.79, 0.90	0.57, 1.00	0.57, 1.00	0.93, 0.95	0.86, 1.00	0.86, 1.00

Q-Statistic quality measure	230.76	210.8	185.38	16.42	140.00	243.06	241.99	254.67	217.23

Number of genes selected	6	17	4	33	7	2	2	70	12

**Table 3:** Maximum performance results on the independent test-set under the first testing scenario Alon et al. [15] (colon cancer) data set.

	**Filter Method**	**Wrapper Methods**					**Hybrid Methods**		
Criterion	Fisher's Ratio	RFE-LNW-GD	RFE-SVM	RFE-LSSVM	RFE-RR	RFE-FLDA	RFE-LNW1	RFE-LNW2	RFE-FSVs-7DK

Accuracy	0.89	0.94	0.89	0.94	0.67	0.89	0.94	0.89	0.94
Sensitivity, Specificity	1.00, 0.67	1.00, 0.83	0.92, 0.83	1.00, 0.83	0.58, 0.83	1.00, 0.67	1.00, 0.83	1.00, 0.67	1.00, 0.83

Q-Statistic quality measure	128.39	127.46	72.74	50.33	33.68	33.76	92.83	128.43	103.39

Number of genes selected	16	9	8	11	10	8	10	16	25

We first notice that for the BC data set (Table 1) RFE-FSVs-7DK is the best accuracy performer, achieving the highest success rate of 95% (only one sample is misclassified) with 73 genes selected. It also achieves the highest specificity measure with adequately high sensitivity. Noticeable is the fact that the same method achieves a relatively high statistical significance score, close to that of the filter method, which is the highest performer of the Q-statistic. RFE-LNW1 also demonstrates noticeable performance both in terms of accuracy and statistical significance. Alternatively, the RFE-LNW2 and filter method have similar results indicating that by using a relatively low learning rate RFE-LNW approach converges to the result of the pure filter method. From the wrapper methods, RR and FLDA achieve high accuracy with better sensitivity than specificity values.

For the leukemia dataset (Table 2) most algorithms (except the wrapper RR and FLDA methods) attain higher accuracy than in breast cancer (Table 1), which is justified due to its well defined classes. The specificity of all methods is remarkably high. Nevertheless, this is not true for sensitivity with only LNW, SVM and hybrid methods achieving scores over 86%. Moreover, the hybrid methods preserve good values of the Q-statistic, slightly better than the filter method, revealing high discrimination over the selected gene signatures. The wrapper methods in general reflect lower values of the Q-statistic metric, implying weaker discrimination of genes amongst the classes of interest, and low correlation between gene expression level and prediction outcome. The wrapper LNW and SVM methods reflect better performance than the filter method.

For the CC data set (Table 3) all methods (except the wrapper RR) achieve more than 88% accuracy, with wrapper LNW-GD, LSSVM and hybrid LNW1 and RFE-FSVs being the best performers, achieving also the highest sensitivity rate. We point out the inferior performance of the wrapper RR method compared to previous results indicating that the specific algorithm is highly affected by differences in the experimental settings, as it is also pointed out in the leukemia data set. The sensitivity achieved by all methods (except RR) is remarkably high while at the same time, the hybrid methods preserve higher values of the Q-Statistic compared to the wrapper schemes and close or better (RFE-LNW2) than the filter method. An exception is the wrapper LNW-GD which achieves a high score close to the filter method as well. Overall, the hybrid schemes preserve top performance amongst the tested methods for all data sets. We may also infer that the wrapper RR and FLDA methods are most affected by the differences in the experimental settings.

#### Cross-validation

The results of this experimental scenario concerning BC, leukemia and CC data sets are presented in Tables 4, 5, 6, respectively. We can observe that filter method perform well in all cases, resulting in higher accuracy and smaller CIs. There is a similar tendency for the hybrid methods for all data sets. For the wrapper methods, however, the performance varies depending on the data set. Furthermore, the wrapper methods derive wider CIs in the more complex set of breast cancer, indicating larger variation of results throughout the CV iterations.

**Table 4:** Maximum Performance results on cross validation under the second testing scenario; 10-fold cross validation Van't Veer et al. [23] (breast cancer) data set.

	**Filter Method**	**Wrapper Methods**					**Hybrid Methods**		
Criterion	Fisher's Ratio	RFE-LNW-GD	RFE-SVM	RFE-LSSVM	RFE-RR	RFE-FLDA	RFE-LNW1	RFE-LNW2	RFE-FSVs-7DK

Accuracy	0.88	0.78	0.76	0.75	0.74	0.75	0.82	0.88	0.85
Sensitivity, Specificity	0.83, 0.90	0.77, 0.81	0.68, 0.80	0.68, 0.80	0.68, 0.77	0.69, 0.80	0.74, 0.88	0.82, 0.90	0.84, 0.86

Confidence Interval	0.21	0.27	0.29	0.28	0.26	0.27	0.25	0.21	0.21

Number of genes selected	35	26	33	36	39	28	35	33	21

**Table 5:** Maximum Performance results on cross validation under the second testing scenario; 10-fold cross validation Golub et al. [9] (leukaemia) data set.

	**Filter Method**	**Wrapper Methods**					**Hybrid Methods**		
Criterion	Fisher's Ratio	RFE-LNW-GD	RFE-SVM	RFE-LSSVM	RFE-RR	RFE-FLDA	RFE-LNW1	RFE-LNW2	RFE-FSVs-7DK

Accuracy	0.99	0.99	0.99	0.99	0.48	0.997	0.96	0.99	0.98
Sensitivity, Specificity	0.95, 1.00	1.00, 0.99	0.95, 1.00	0.98, 0.99	1.00, 0.31	0.99, 1.00	0.90, 0.98	0.95, 1.00	0.91, 1.00

Confidence Interval	0.1	0.08	0.1	0.09	0.4	0.05	0.17	0.1	0.12

Number of genes selected	4	5	4	30	6	5	4	4	3

**Table 6:** Maximum performance results under the second testing scenario; 10-fold cross validation Alon et al. [15] (colon cancer) data set.

	**Filter Method**	**Wrapper Methods**					**Hybrid Methods**		
Criterion	Fisher's Ratio	RFE-LNW-GD	RFE-SVM	RFE-LSSVM	RFE-RR	RFE-FLDA	RFE-LNW1	RFE-LNW2	RFE-FSVs-7DK

Accuracy	0.90	0.87	0.87	0.91	0.83	0.89	0.91	0.89	0.91
Sensitivity, Specificity	0.92, 0.88	0.89, 0.85	0.92, 0.79	0.97, 0.81	0.77, 0.91	0.93, 0.84	0.93, 0.88	0.93, 0.84	0.93, 0.89

Confidence Interval	0.25	0.29	0.29	0.22	0.31	0.26	0.22	0.26	0.24

Number of genes selected	16	17	16	22	19	14	10	15	12

For the CV testing of BC, the wrapper schemes exhibit less accuracy and stability, yielding larger CIs. The inferior accuracy of wrapper methods is primarily attributed to their lower sensitivity. Alternatively, for the leukemia data set (Table 5) most wrapper schemes (except RR) increase their maximum accuracy measures in CV compared to independent test-set scenario, whilst attaining good sensitivity and specificity scores. In fact, the FLDA scheme reaches the maximum accuracy followed by LSSVM and LNW-GD, but in this case the accuracy performance of all methods is comparably high.

For the CC data set (Table 6), even though all methods decrease their performance in comparison to leukemia data set, they still exhibit a high accuracy level ranging from 83% to 91%. We point out the increase in the performance of the wrapper RR method, compared to the previous evaluation procedure (independent test-set scenario), indicating once more that the method is strongly influenced by the different experimental scenarios. As in the case of the leukemia data set, a decrease in algorithmic performance is observed when compared to the independent test-set scenario. The wrapper LSSVM and hybrid FSVs and LNW1 are the top performers, with LSSVM achieving a higher sensitivity rate among the three. Generally speaking, the wrapper methods (with the exception of LSSVM and FLDA methods) have higher CIs than the hybrid and the filter methods, indicating higher variation of results throughout the CV iterations, as was also expressed in BC data. Moreover, hybrid methods tend to select less number of genes than both the filter and wrapper approaches.

Considering these results, we may conclude that the performance of pure wrapper methods is more affected by the experimental conditions with performance measures varying over cross-experimental evaluation. Furthermore, wrapper methods appear to benefit from integrating appropriately adapted filtering criteria (hybrid approaches) into their learning procedure. Such integration would lead to a more stable performance by preserving high levels of statistical significance under both tested scenarios and experimental frameworks.

Overall, the evaluation (or ranking) of algorithms changes under a cross experiment evaluation or when considering different evaluation schemes such as CV or single-step evaluation with testing on an independent test-set. The CV scheme appears to give a certain (positive or negative) bias to all algorithms. Furthermore, the optimal size of the selected gene-set appears to be dependent on the evaluation scheme. The CV scheme driven by its various training sets can lead to a quite skewed distribution of performance estimates for various sizes of selected gene signatures. Thus, the final size of signature attaining maximum performance within the multiple iterations of CV can be easily affected by several random effects. In essence, factors of bias in maximum CV performance include the small size correlation effect and the intermixing of samples within the training and test-set. Similar criticism on maximum or ranked performance schemes has been reported by other studies [24].

From this section it is obvious that the ranking of methods is highly dependent on the evaluation strategy. For a more objective comparison of algorithms, we consider in the next section the average performance of algorithms at a certain cut-off point on the number of surviving genes. Thus, the size of the gene signature is specifically chosen for each algorithm based on its best performance on the independent test-set (Tables 1, 2, 3), in order to test how well algorithms will perform with new cases.

### Average Performance Results on Cross-validation

The average per run and per subject accuracies (*acc*_*R *_and *acc*_*P*_) along with their standard deviations (stds) are tabulated in Tables 7, 8, 9. The measures of sensitivity and specificity are also reported on the basis of per run tests for the test population in CV and in the independent sets. The algorithms achieving the highest accuracy as well as the lowest stds are highlighted with bold figures. The last row of those tables presents the overall measures on all samples tested (CV test set plus the independent test set) and reflects the overall performance ability of each algorithm. When considering the same population for testing, e.g. the independent test-set, the two measures, *acc*_*R*_^*t *^(per run) and *acc*_*P*_^*t *^(per subject), obtain identical values. Notice, however that we expect a difference in stds due to the different reference cohorts, i.e. the different runs and subjects used.

**Table 7:** Average performance measures under the third testing scenario, sample mean and std of accuracies Van't Veer et al. [23] (breast cancer) data set.

	**Filter Method**	**Wrapper Methods**					**Hybrid Methods**		
Method	Fisher's Ratio	RFE LNW-GD	RFE-SVM	RFE-LSSVM	RFE-RR	RFE-FLDA	RFE-LNW1	RFE-LNW2	RFE-FSVs-7DK

Number of genes selected	61	22	32	45	7	28	44	64	73

Per Subject AccP-CV	**0.744**	0.612	0.580	0.644	0.509	0.573	0.612	**0.788**	0.668
Std(P)-CV	0.320	0.306	0.354	0.385	***0.267***	0.276	0.352	***0.254***	0.387

Per Subject AccP-Test	0.660	0.719	0.584	0.658	0.659	0.614	**0.753**	0.669	**0.735**
Std(P)-Test	0.230	0.277	0.294	0.358	***0.173***	0.240	0.236	***0.217***	0.221

Per Run AccR-CV	**0.708**	0.587	0.570	0.598	0.514	0.546	0.621	**0.708**	0.653
Sensitivity, Specificity	**0.66, 0.70**	0.53, 0.63	0.54, 0.61	0.43, 0.61	0.50, 0.58	0.52, 0.56	0.55, 0.67	**0.67, 0.73**	0.64, 0.63
Std(R)-CV	0.174	0.172	0.187	0.175	0.196	0.199	***0.156***	***0.158***	0.164

Per Run Test AccR-Test	0.660	0.719	0.584	0.658	0.659	0.614	**0.753**	0.669	**0.735**
Sensitivity, Specificity	0.65, 0.64	**0.70, 0.75**	0.63, 0.59	0.55, 0.69	0.69, 0.50	0.65, 0.55	**0.79, 0.68**	0.67, 0.67	**0.76, 0.55**
Std(R)-Test	0.102	0.099	0.085	***0.077***	0.102	0.116	***0.073***	0.104	***0.079***

Per Run All AccR-Overall	0.673	0.684	0.580	0.642	0.620	0.596	**0.717**	0.680	**0.713**
Std(R)-Overall	0.091	0.083	0.080	0.080	0.098	0.105	***0.068***	0.087	***0.077***

**Table 8:** Average performance measures under the third testing scenario, sample mean and std of accuracies Golub et al. [9] (leukaemia) data set.

	**Filter Method**	**Wrapper Methods**					**Hybrid Methods**		
Method	Fisher's Rattio	RFE-LNW-GD	RFE-SVM	RFE-LSSVM	RFE-RR	RFE-FLDA	RFE-LNW1	RFE-LNW2	RFE-FSVs-7DK

Number of genes selected	6	17	4	33	7	2	2	70	12

Per Subject Acc(P)-CV	**0.95**	0.94	0.88	0.73	0.42	0.89	0.93	**0.97**	0.93
Std(P)-CV	**0.15**	0.20	0.21	0.40	0.39	**0.16**	0.23	**0.16**	0.21

Per Subject Acc(P)-Test	0.87	0.86	0.82	0.78	0.70	0.81	**0.91**	**0.88**	0.85
Std(P)-Test	0.25	0.28	0.30	0.31	**0.19**	0.23	**0.2**	0.26	0.28

Per Run Acc(R)-CV	**0.96**	0.94	0.89	0.75	0.39	0.89	0.93	**0.97**	0.94
Sensitivity, Specificity	0.87, 0.99	0.89, 0.96	0.65, 0.97	0.47, 0.85	0.99, 0.19	0.74, 0.94	0.88, 0.95	0.89, 0.99	0.69, 0.98
Std(R)-CV	**0.09**	0.107	0.14	0.20	0.12	0.16	0.14	**0.09**	0.11

Per Run Test Acc(R)-Test	0.87	0.86	0.82	0.78	0.70	0.81	**0.91**	**0.88**	0.85
Sensitivity, Specificity	0.69, 1.00	0.67, 0.99	0.58, 0.98	0.62, 0.90	0.57, 0.79	0.60, 0.95	0.87, 0.94	0.72, 0.99	0.52, 0.95
Std(R)-Test	**0.03**	0.05	0.07	0.06	0.11	0.08	**0.04**	0.05	0.05

Per Run All Acc(R)-Overall	0.88	0.87	0.82	0.78	0.67	0.82	**0.91**	**0.89**	0.86
Std(R)-Overall	**0.04**	0.05	0.06	0.05	0.10	0.08	**0.04**	**0.04**	0.05

**Table 9:** Average performance measures under the third testing scenario; sample mean and std of accuracies Alon et al. [15] (colon cancer) data set.

	**Filter Method**	**Wrapper Methods**					**Hybrid Methods**		
Method	Fisher's Ratio	RFE-LNW-GD	RFE-SVM	RFE-LSSVM	RFE-RR	RFE-FLDA	RFE-LNW1	RFE-LNW2	RFE-FSVs-7DK

Number of genes selected	16	9	8	11	10	8	10	16	25

Per Subject Acc(P)-CV	**0.77**	0.70	0.71	0.74	0.65	0.73	0.74	0.74	**0.75**
Std(P)-CV	0.32	0.31	0.34	0.40	**0.30**	**0.29**	0.31	0.32	0.32

Per Subject Acc(P)-Test	0.82	0.79	0.80	**0.90**	0.67	0.79	0.80	0.81	**0.85**
Std(P)-Test	0.26	0.26	0.26	0.23	**0.22**	**0.22**	0.29	0.25	0.24

Per Run Acc(R)-CV	**0.77**	0.70	0.72	0.72	0.66	0.74	0.75	0.75	**0.76**
Sensitivity, Specificity	0.81, 0.73	0.75, 0.63	0.80, 0.60	0.86, 0.51	0.60, 0.76	0.79, 0.66	0.76, 0.75	0.8, 0.68	0.77, 0.73
Std(R)-CV	0.19	0.19	**0.18**	**0.17**	0.19	0.18	0.19	0.20	0.19

Per Run Test Acc(R)-Test	0.82	0.79	0.80	**0.90**	0.67	0.79	0.80	0.81	**0.85**
Sensitivity, Specificity	0.94, 0.58	0.88, 0.63	0.92, 0.58	0.98, 0.73	0.61, 0.80	0.88, 0.60	0.91, 0.58	0.93, 0.59	0.95, 0.64
Std(R)-Test	0.08	0.09	**0.07**	0.10	0.13	0.08	0.08	0.08	**0.06**

Per Run All Acc(R)-Overall	0.81	0.77	0.79	**0.86**	0.67	0.78	0.79	0.81	**0.83**
Std(R)-Overall	0.08	0.09	**0.06**	0.09	0.11	0.08	0.07	0.08	**0.06**

**Figure 2:**
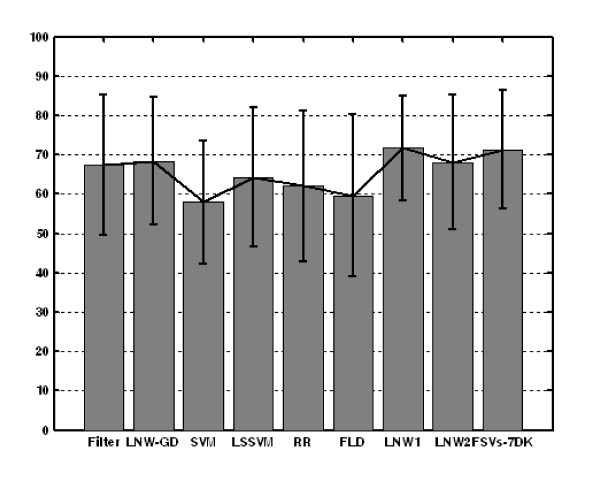
**Performance evaluation in breast cancer**. Average cross validation performance and confidence intervals of algorithms for the breast cancer data set of [23].

**Figure 3:**
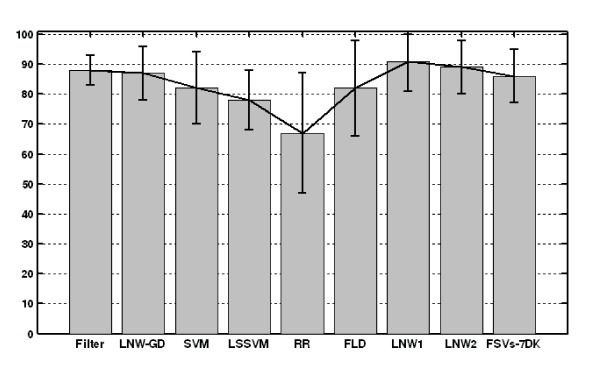
**Performance evaluation in leukaemia**. Average cross validation performance and confidence intervals of algorithms for the leukemia data set of [9].

**Figure 4:**
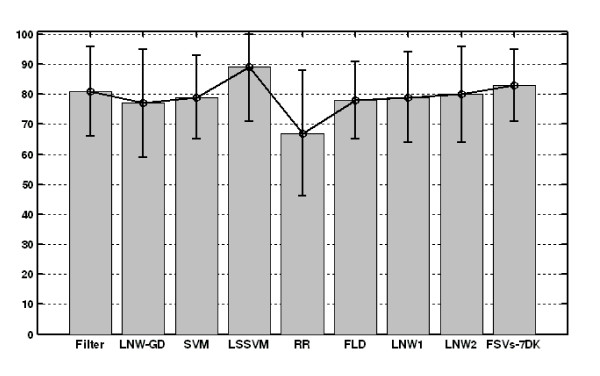
**Performance evaluation in colon cancer**. Average cross validation performance and confidence intervals of algorithms for the colon cancer data of [15].

**Figure 5:**
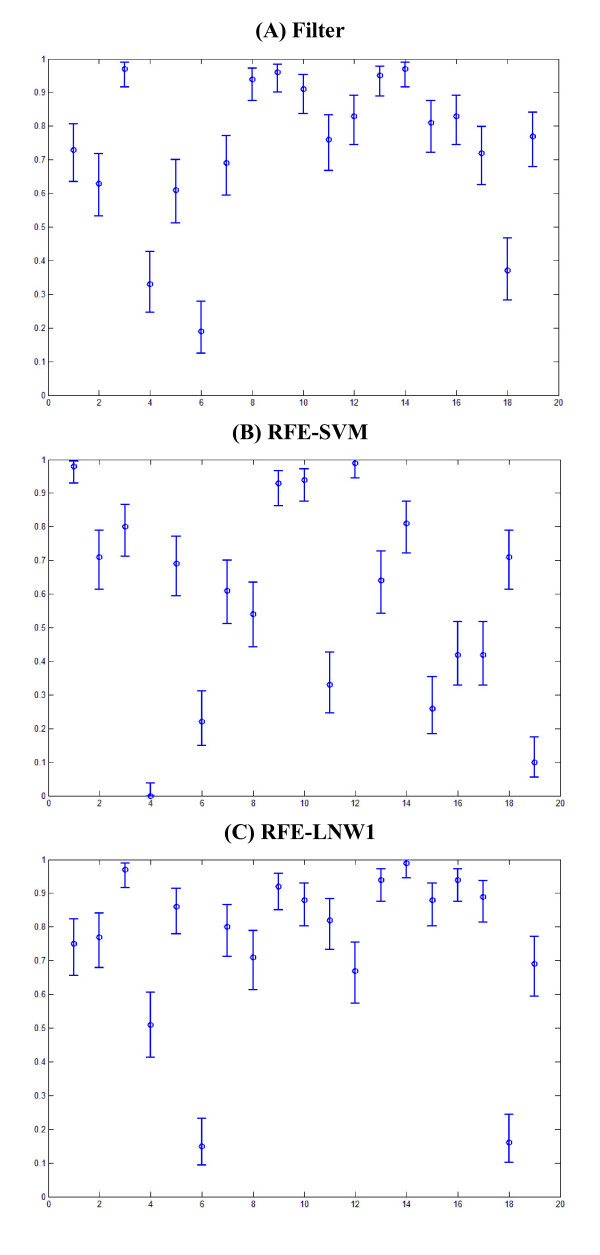
**Per-subject performance evaluation and confidence intervals in breast cancer**. Average cross validation accuracies and confidence intervals per-subject, in the independent test-set of breast cancer data [23]. 19 subjects tested 100 times for the (a) filter, (b) wrapper SVM and (c) hybrid LNW1 algorithms.

**Figure 6:**
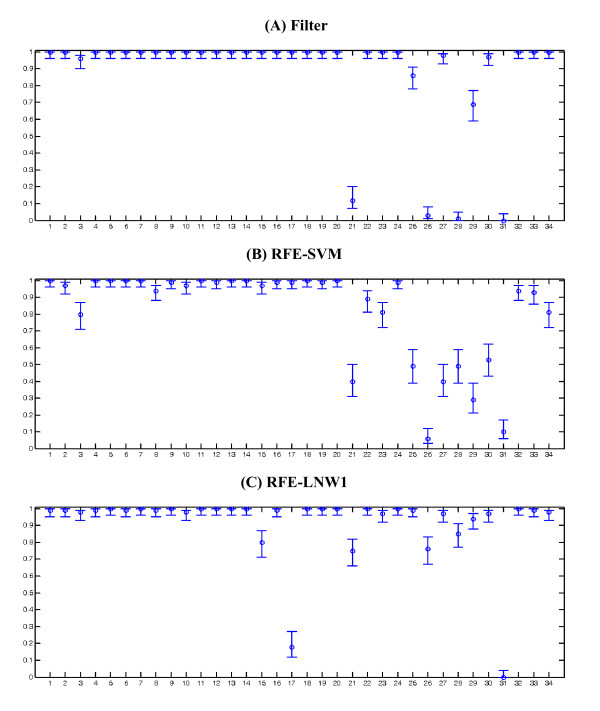
**Per-subject performance evaluation and confidence intervals in leukaemia**. Average cross validation accuracies and confidence intervals per-subject in the independent test-set for leukemia data of [9]. 34 subjects tested 100 times for the (a) filter, (b) wrapper SVM and (c) hyprid LNW1 algorithms.

**Figure 7:**
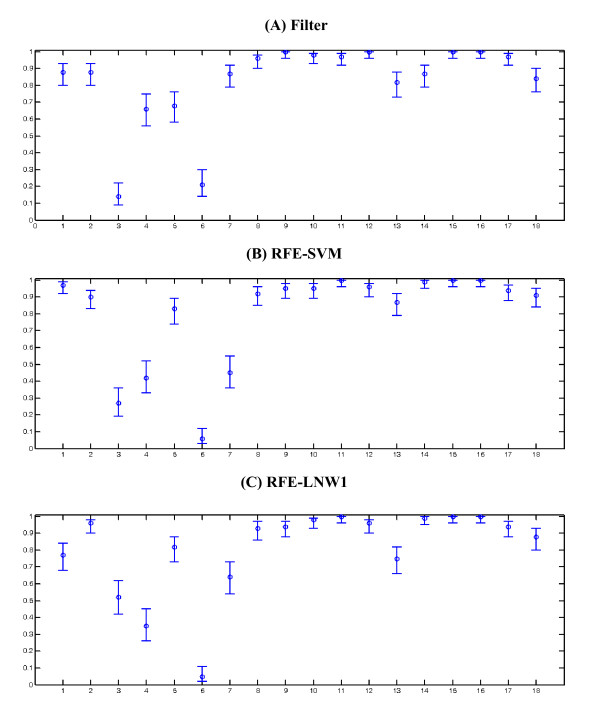
**Per-subject performance evaluation and confidence intervals in colon cancer**. Average cross validation accuracies and confidence intervals per-subject in the independent test-set for colon cancer data [15]. 18 subjects tested 100 times for the (a) filter, (b) wrapper SVM and (c) hyprid LNW1 algorithms.

The overall accuracy measures along with their CIs are graphically depicted in Figures 2, 3, 4. Furthermore, Figures 5, 6, 7 show the per subject accuracy and the CIs of classification accuracy for the independent test-sets. Note the large variability among sample accuracies in the case of BC (Figure 5) and the relative consistency of estimation throughout the tested subjects in the case of leukemia (Figure 6). Concerning the consistency of algorithms in terms of selected gene signatures over the CV iterations, the consistency (or gene overlap) index is tabulated in Table 10 for all tested algorithms. With the exception of the LSSVM and RFE-LNW-GD methods, wrapper methods appear to select different genes per iteration, resulting in quite small indices. Filter, as well as hybrid, methods yield good consistency based on their high frequencies of selecting the same genes throughout CV iterations. Nevertheless, we should stress our belief that in a further development stage we need to also associate the statistical results with the biological meaning of selected gene signatures.

**Table 10:** Consistency index (average gene frequency) for the tested algorithms.

**Method-Group Kategory Kategory**	**Method**	**Breast Cancer**	**Leukemia**	**Colon Cancer**
Filter Method	Fisher's ratio	**0.64**	**0.74**	**0.80**

Wrapper Methods	RFE-LNW-GD	**0.70**	0.71	0.65
	
	RFE-SVM	0.49	0.37	0.35
	
	RFE-LSSVM	**0.87**	**0.89**	**0.87**
	
	RFE-RR	0.18	0.06	0.30
	
	RFE-FLDA	0.32	0.25	0.22

Hybrid Methods	RFE-LNW1	0.53	0.63	0.55
	
	RFE-LNW2	**0.64**	**0.78**	**0.79**
	
	RFE-FSV-7DK	0.58	0.63	0.63

Comparing the performance results, we first notice that the average accuracy measures (Tables 7, 8, 9) are significantly smaller than their maximum counterparts in the previous section (Tables 1, 2, 3). Recall that in the present scenario each algorithm is applied with fixed parameters for all CV iterations and terminates at a fixed number of surviving genes, which is specified by its point of maximum performance on the independent test-set (Tables 1, 2, 3). This scenario is much closer to a clinical testing and validation set-up, so that these results, even though inferior, reflect more closely the actual potential of algorithms in clinical prediction. It can also be observed that, similarly to the maximum performance measures, the average accuracy of the CV samples differs from that of the independent test samples, either on a per run or a per subject basis (Tables 7, 8, 9). This deviation is consistent for all three datasets. Furthermore, all methods demonstrate consistently lower stds when tested on the independent test-set than on the testing fold of the CV set for the BC and CC data sets (Tables 7 and 9). This is a direct consequence of the fact that in the former case the testing set is kept constant across all iterations (fixed independent test-set). Nevertheless, this performance is reversed for the leukemia data set (Table 8), which is possibly due to statistical differences in the distributions of the training (CV) and testing samples and implies insufficient training of the algorithms.

Considering the results over all testing samples (Tables 7, 8 and 9), which we consider as good estimates of the true algorithmic performances (golden standard for comparison), we notice that they are more closely approximated by the results of the independent test-set than by those of CV test folds. More specific comparisons on algorithmic performance are summarized in the following sections.

#### Filter and Wrapper methods

All methods reflect a positive or negative bias on the accuracy estimates of the CV set over those of the independent test-set and the overall testing set, depending on the data set. The baseline filter method for all data sets derives good accuracy on the CV tests (Tables 7, 8, 9), which is in accordance to the results of [6]. In fact, its accuracy on CV tests is higher than all wrapper methods tested. Nevertheless, such a good performance is not sustained on the independent test-set (Tables 7, 8, 9). Furthermore, the std indices for the leukemia data set achieve their lowest values for the filter method, but such algorithmic stability is dependent on the data set.

The wrapper scheme based on the linear neuron (LNW-GD) results in better accuracy on the independent test-set, either on a per run or per subject basis (Tables 7 and 9) for the more difficult BC and CC data sets. This is reversed for the leukemia data set (Table 8), where the compact nature of samples in each class forces its performance on CV testing towards overestimated measures. Nevertheless, it maintains reasonable performance on the overall test-set, with balanced sensitivity and specificity.

Considering the BC data set and the other wrapper RFE approaches on their average per run performance, we observe that on the independent test-set (Table 7) RR and FLDA perform better than SVM, which is in accordance to the results of [8]. However, the SVM and its least squares variant yield better specificity than the RR and FLDA methods. This performance is reversed when considering CV (Table 7). In this case, the performance of SVM is slightly better than FLDA, which is in accordance to the findings of [5]. For the leukemia data set (Table 8) the SVM-based approaches perform better for both the independent test-set and the CV approaches, with increased specificity, but reduced sensitivity over the RR and FLDA methods. This improvement is attributed to the characteristic distribution of support vectors in the leukemia data set, which expresses better class separability compared to the BC data set [32]. Similarly for CC (Table 9), the SVM-based approaches perform better than the other wrapper methods, but now due to increased sensitivity. With the exception of the excellent performance of the LSSVM on the independent and the overall test-sets, the performance of wrapper methods in colon cancer is in accordance to the performance ranking for the other two data sets.

Despite its high performance in CC, the LSSVM method expresses a large variation on the accuracy measures for CV testing and the independent test-set, which may be due to the particular selection of the independent test-set. Notice that in this case the independent set was randomly selected from the original data in [15], unlike the other two data sets considered which supplied different independent data sets. Furthermore, its increased performance on the independent test-set is mainly due to its increased sensitivity over all other algorithms (Table 9). The sporadic nature of its results is also verified by its rather low performance on the other two data sets. Overall, for all data sets the variation of results in wrapper methods is quite large between the CV and the independent test-set data sets, indicating a significant influence of the training and/or testing sets on the performance of these algorithms. In particular, the LSSVM achieves its best performance on colon cancer. On similar grounds, the RR method performs relatively similar to the other wrapper methods on breast cancer, but presents the worst performance on the other two data sets. These results further highlight the need for evaluating the ranking of wrapper algorithms on a series of tests over different data sets. An additional drawback for wrapper methods is the weak consistency of the derived gene signatures between iterations, as reflected by their low consistency index presented in Table 10. Only the LSSVM approach attains exceptionally high consistency index, indicating a consistent selection of genes, but the selected gene signatures in most cases do not reflect high prediction power. Hybrid and filter methods combine relatively high consistency index with increased success rate and more stable performance over the different datasets.

#### Hybrid methods

Concerning the hybrid approaches, the FSV scheme is one of the best two algorithms in BC and CC, with its performance being slightly inferior of other hybrid methods in the leukemia data set. More specifically, for the BC and CC case FSV succeeds high accuracy on either a per run or a per subject base when testing the independent and the overall test-set (Table 7 and 9). This performance slightly drops relative to other hybrid methods in the case of leukemia, where the CV scheme reflects highly overestimated measures (Table 8). A possible reason for that is that the optimal representation kernel (7 degree polynomial) of the BC data set is also used for the leukemia and CC data sets. A different kernel with better fit to the distribution of the leukemia or CC data sets would further improve the performance of this method.

The neural network-based algorithms (LNW1 and LNW2) are amongst the best performers in BC and leukemia data sets. The LNW2 algorithm exhibits highly optimistic estimates in CV (Tables 7, 8), while its overall performance is amongst the highest for leukemia. The LNW1 approach yields good per run accuracies for these data sets, when tested on either the independent test-set (Tables 7 and 8) or the entire test-set (last row in Tables 7 and 8). It also derives consistently small variances per run, reflecting good stability properties as it appears to be less affected by variation of the training and/or testing sets. The performance rank of LNW1 and LNW2 on CC drops slightly, mainly due to the exceptional performance of the wrapper LSSVM method. Nevertheless, all hybrid methods perform better than RFE-LSSVM on cross validation (Table 9), while they all achieve above 80% accuracy on the independent test-set (Table 9) with the best performer being the RFE-FSVs (85% average success rate).

Testing on all available samples (both sampling and independent test-sets) through CV is regarded to be a more unbiased test on algorithmic performance and is used as a reference for comparison (Tables 7, 8, 9). Regarding such overall performance results, the hybrid FSV approach takes the lead in overall performance, followed by LNW1 and LNW2, with slight variations.

The performance of hybrid methods on the overall set is closely matched by the results of testing on the independent test-set throughout the iterations, either on per run or per subject basis, further emphasizing the necessity of an independent test-set in evaluation procedures and the utility of both performance and stability measures. These hybrid methods also achieve relatively high consistency index compared to wrapper methods (except the LSSVM) as illustrated in Table 10. Thus, considering several aspects of their performance, we may claim that the hybrid approaches provide consistently good performance and stability in the independent test-set, both on per run and per subject basis.

#### Outlier Samples

Another important aspect of this work is the consideration of the influence of noisy samples. Considering the per subject stability (std measure) of algorithms in all cases, we observe relatively increased values compared to the per run measures (Tables 7, 8 and 9), which indicate a quite large variability in the algorithmic estimates for individual case depending on the training set. This is an indication of problems caused by the limited training-set used in the design of prediction models. Since the model is designed with limited information from the feature space, specific test samples may not fit well to its design specifications. The variability for the independent test-set is graphically presented in Figures 5, 6, and 7 for the baseline filter, wrapper SVM and hybrid LNW1 approaches and the three cases, respectively. An accuracy deviation on specific samples is more severe in breast cancer and less severe in leukemia, following the difficulty in class separability illustrated by the PCA analysis in Figure 1.

Further analyzing the BC case (Figure 5), we notice that the performance of algorithms on test samples is variable, with the exception of some samples on which the average performance of all algorithms is quite low. These specific samples are ID 37, 38, 54, 60 and 76 from the Van't Veer's training-set, as well as sample ID 117 from the independent test-set. Generally, these samples cannot be well classified and can be characterized as outliers. When excluding only these samples, the average performance measures are significantly affected, as presented in Table 11. Comparing the corresponding measures in Tables 7 and 11, it becomes obvious that only a few outliers due to measurement errors can drastically deteriorate the performance of any prediction algorithm.

**Table 11:** Average performance measures on the Van't Veer et al. [23] (breast cancer) data set after removing samples suspected for measurement noise.

	**Filter Method**	**Wrapper Methods**					**Hybrid Methods**		
Criterion	Fisher's Ratio	RFE LNW-GD	RFE-SVM	RFE-LSSVM	RFE-RR	RFE-FLDA	REF-LNW1	REF-LNW2	RFE-FSVs-7DK

Number of Genes	61	22	32	45	7	28	44	64	73

Per Subject Acc(P)-CV	0.80	0.71	0.69	0.76	0.59	0.65	0.74	0.88	0.78
Per Subject Acc(P)-Test	0.68	0.74	0.62	0.68	0.68	0.64	0.77	0.69	0.76

Per Run Acc(R)-CV	0.74	0.63	0.62	0.65	0.55	0.576	0.69	0.74	0.70
Per Run Test Acc(R)-Test	0.68	0.74	0.62	0.68	0.68	0.641	0.77	0.69	0.76

Per Run All Acc(R)-Overall	0.69	0.71	0.62	0.67	0.65	0.625	0.74	0.70	0.74

All measures are significantly increased compared to those of Table 7 with the removal of only a few samples.

In a similar consideration for the leukemia data set (Figure 6), we may conclude that sample 31 is probably an outlier since all methods examined fail to classify it. Furthermore, for CC (Figure 7) we may suspect samples 3, 4 and 6 as outliers, since most methods fail to effectively classify them. The identification and removal of such samples is of primary importance in algorithmic evaluation, especially in the area of gene selection with sparsely covered data spaces. The identification scheme proposed here is an alternative to data projection for exploratory purposes [33] for selecting outlier samples based on machine learning rather than on projective mappings of data distributions.

## Discussion

In this study data-driven models, which highly depend on the distribution of data within and across classes, were considered. The cut-off point on the size of gene signature selected was determined independently for each model, based on its maximum performance on the independent test-set. Even though the ranking of algorithms tested varied between data sets, the main focus was on methodological aspects of evaluation so that the points addressed would remain valid for other data sets and algorithms.

More specifically, the examined approaches were applied in three different data sets of gradual difficulty as a first attempt to understand the various CV approaches and form the basis of as an objective as possible algorithmic evaluation scheme. The BC data set, which had the less well defined classes, and its independent test-set was used as a pilot in the evaluation process. Each of the methods examined was pushed to perform its best level on the independent test-set of the pilot data set, by appropriately fine tuning its parameters. Then, using exactly the same set of parameters, method performance was assessed, using various metrics in two additional data sets, along of course with the pilot one. Using such an approach we aimed in assessing the sensitivity of each method in various data sets or different biomedical experimental scenarios. This approach revealed that RFE-RR and RFE-LSSVM for instance, highly depend on the data set by producing diverse and contradictory results along the different computational scenarios presented.

Three different evaluation schemes were used. The maximum accuracy scheme, tested on both CV and independent test-sets, presented optimistic results for most algorithms and all data sets considered. Such results can be misleading and induce severe bias on the accuracy estimates for predictors, which are far away from the actual potential of each algorithm on correctly classifying new unseen cases. The use of such schemes should be avoided and the average accuracy scheme should be used instead.

Following these guidelines, the performance of all methods was assessed with a 10-fold evaluation process on the specific number of genes pointed by the independent test-set evaluation. This strategy aimed in revealing the consistency of each method in deriving a gene signature with high prediction accuracy, either on per run or per patient basis. The former assessed method sensitivity on perturbations of the test-set, while the later addressed sensitivity on the training set. This evaluation scenario revealed that the use of a stable independent test-set along with a 10-fold evaluation process (that uses a different training set per fold) resulted in more stable and less variant method performance, than a standard 10-fold CV process. This result was verified in all three data sets on the per-run basis, while on the per-patient basis it was verified for two data sets (BC and CC). We found that complementing a stable test-set with a varying one along a 10-fold CV process is a less unbiased estimator of method performance on the initial independent test-set than a standard 10-fold CV process.

Using the per-patient approach we identified outlier samples, which usually resulted in an under-estimation of method performance, while stability index was used to access method consistency along the iterative 10-fold CV-process. Such an index however, should be used with caution and always in association with the accuracy performance.

Overall, the wrapper methods expressed large variations in performance depending on the data set. The filter method derived good results for all data sets on maximum performance test, but its performance ranking dropped when average accuracy testing was considered, especially for the independent test-set. The hybrid methods preserved consistently good results for either maximum or average performance consideration and for all data sets tested. Focusing on the average performance results in CV testing, the filter method was among the highest performers along with hybrid approaches. On the independent test-set and the overall testing scheme, which forms the golden standard for our comparisons, the best performers always included FSV and other hybrid methods, except in the last data set where LSSVM yielded relatively high accuracy.

It becomes clear that comparison and ranking of algorithms, even on the same data set, should be based on several characteristic measures. Furthermore, the relatively small overlap index raises concerns regarding the potential influence of the small sample random correlation effect on the derived signatures and the performance estimates. A reason for such effects is the independent selection process throughout the iterations for the different data folds. Under CV, each iteration begins with the maximum number of genes and recursively eliminates them up to the minimum specified size, with its own ranking scheme driven by the data in the training set. Thus, for a specific size gene signature, the process for each iteration may select completely different genes. Such drawbacks of CV have been highlighted before, suggesting the need for double CV for meta-parameter selection [34]. For the purpose of gene signature selection, we propose the use of a nested CV scheme, where all iterations operate in parallel. At every cut-off stage on the size of gene signature, all genes could be ranked based on the average of their weights from all CV iterations, so that the same genes survive for all iterations. In this way the effect of random correlation between gene-sets and data samples is expected to be reduced. This scheme, however, remains to be tested in practice.

## Conclusion

The prediction accuracy reported by many studies in the field of microarray analysis reflect a certain bias due to either the study design, the analysis method (model design) or the validation process [35,16]. Most published comparative studies consider that issue as well as the ranking of algorithms on various data sets. This study has focused in the evaluation platform of algorithmic performance. The main aim was not to strictly compare algorithms, even though a ranking was attempted as a byproduct of this work, but rather to address inefficiencies of evaluation and introduce aspects that may reduce uncertainty in algorithmic evaluation.

Overall, it is concluded that the use of an independent test-set is beneficial for estimating a baseline on accuracy results for all algorithms. The CV scheme by itself induces certain positive or negative bias depending on the data set and should be complemented with independent tests. Nevertheless, most often data sets do not come with a compatible independent test-set obtained under the same study criteria. Hence, the isolation of a subset as an independent test-set must be considered with caution, since there is a danger of inducing estimation bias due to alterations in the design of the experiment. Furthermore, to reduce bias in the estimation of measures, the design of cross-validation splits should be carefully considered as it should not alter the entire design of the experiment. In particular, a stratified scheme for sample selection should be preferred, so that individual samples can be tested a sufficient number of times throughout the iterations.

Another concept that has been introduced is the "performance profile", which is a matrix recording accuracy results over the various iterations (for different CV folds) of the RFE process and enables the computation of subject and iteration-specific performance measures for each algorithm. Based on the performance profile, we consider case-specific measures to reveal stability of the estimate over different training sets, as well as frequency measures on gene selection to address the algorithm's consistency in selecting the same gene signature under different training conditions. These issues reveal different aspects of algorithmic performance which could influence their ranking under a cross-validation strategy.

Besides all these algorithmic considerations and comparisons, gene selection should always be interpreted from the biologists' perspectives. Statistical significance is not always accompanied by biological relevance, thus knowledge of gene function and biological pathways should always be taken into account. It seems that a more integrated scheme of statistical analysis, combined with statistical as well as biological validation is needed in order to eliminate any misclassifications and thus could safely being used in the clinical practice and decision-making.

## Methods

### Considerations of the Application Field

Even though gene selection initially appears as a standard paradigm of feature selection, the application domain entails several aspects that add certain constraints to the problem [17,36]. In such an application we have to deal with many problems including the following:

i) *Noise of the data: *DNA microarrays provide a vast amount of data which might contain noise along with redundant information that needs to be processed in such a way so that the real valuable and useful information is finally distilled. This resulting gene signature could then be used by an expert to search, discover and understand the hidden biological mechanisms involved in the development of cancer.

ii) *Limited number of samples*: Most of the microarrays experiments have few samples because of the cost of the method and the limited number of cases in a short study period that adhere to the study protocol. As the number of variables studied is too large relative to the number of cases, *overfitting *can easily occur. A related problem refers to the small sample random correlation effect, which essentially allows a small number of features to be randomly correlated with the data, with an increased risk of getting an irrelevant or random solution,

iii) *Random measurement effects on samples*: each subject may be measured with different (unknown) CIs on each gene expression. Thus, average classification measures and CIs may not be applicable to individual samples; they might apply on gene space distributions but not on subject-specific distributions.

iv) *Bias on the design of the study*: altering the initial number of genes or the number of cases, may change the design conditions of the study. Several studies begin with a smaller set of genes obtained from oversimplified criteria and proceed with the proposed gene elimination approach. However, by coupling different selection methods at the various stages of recursive elimination, one may bias algorithmic performance by means of affecting the initial conditions of recursions.

Taking into consideration the above issues and using the three experimental scenarios presented before, we address several concerns regarding algorithmic evaluation and discuss potential measures for the objective comparison of gene-selection approaches.

### Tested Methods

#### Gene Selection Approaches

In order to select a specific number of features that reflects best performance in sample classification, two general methods exist, namely *filter *and *wrapper *methods. *Filter *methods directly rank genes according to their significance using various statistical measures such as Fisher's ratio, t-statistics, *χ*^2^-statistic, information gain, Pearson's correlation and many others. The top ranked genes that yield the highest classification accuracy are then selected as the final set of markers. *Wrapper *and/or *hybrid *(or integrated) methods employ a classifier in order to assess the importance of genes in decision-making and assign weights to genes by means of the weights of a classifier trained on the data set. Subsequently, the lowest weighted genes are eliminated on the basis of RFE and the process continues in a recursive manner. In the RFE procedure any classifier could be potentially used as a weight vector estimator, highlighting the intriguing advantage of an open and adaptive scheme. The vast majority of methods employ linear predictors for the specific problem of marker selection, due to the sparse nature of the feature space [10]. A fundamental attribute of the "philosophy" of wrapper methods is that gene weights are re-evaluated and adjusted dynamically from iteration to iteration, while in filter approaches gene weights remain fixed. Another qualitative difference between the two philosophies is that filter methods focus on intrinsic data characteristics neglecting gene interactions, while wrapper methods focus on gene interactions neglecting intrinsic data characteristics [19]. Recent studies [26,37] also addressed the advantages of integrating these quite different "philosophies" into a single approach, leading to the so-called hybrid (or integrated) approaches.

Let ***v ***be a particular vector sample of gene values with class label *y*. A linear classifier can be seen as a predictor of the target result *y *that bases its decision on a weight vector ***w ***in the form of $\hat{y}=w^{\prime }v$. In essence, this weight vector identifies the boundary hyperplane between two classes of interest. Based on this formulation, we can use primary characteristics of classifiers used in the various tested methods to assign feature (gene) weights. SVM [38] searches for the boundary hyperplane that provides the best separation distance between classes. It minimizes the regularized power of the weight vector subject to the condition of correct classification of the training set. Least squares support vector machines (LSSVM) [39] modify the above problem into a least squares linear problem. The ridge regression classifier (RR) uses the classification error as a regularizing factor on the minimization of the power of the weight vector [8], whereas Fisher's linear discriminant analysis (FLDA) defines the weight vector as the direction of the projection line of the m-dimensional data that best separates the classes of interest according to the Fisher criterion. The gradient descent linear neuron (LNW-GD) optimizes the *l*_2 _norm of the classification error proceeding in an iterative way imposed by its gradient.

The hybrid/integrated methods considered proceed in a similar iterative manner, but appropriately enrich their learning process within the wrapper scheme with a filter criterion. The linear neuron weight (LNW) approach updates the weight of each individual gene towards the signed direction of the gradient weighted by a factor that induces the effect of the Fisher's metric [26,37]. The learning rate (or update parameter) may be used to control the importance of the Fisher's metric on the derived gene signature. Thus, we test two learning rates along with this method. More specifically, LNW2 uses smaller learning rate than LNW1, favoring the derivation of more statistically significant genes and the creation of more compact classes in the space of surviving features. In a similar framework, the Fisher's support vectors (FSV) approach appropriately integrates a variation of the Fisher's ratio within the weight update scheme of SVMs [26]. By exploiting the kernel formulation of SVM we can derive several forms of the boundary hyper-surface. These recursive methods are compared with the baseline filter method of [9], which uses a variation of Fisher's ratio.

#### Classification Models

The evaluation of feature selection schemes is often performed in association with a subsequent classification model [5,34]. In our study, the classifier models used for testing are obtained from the same pool of methods used for feature selection. This pool covers a wide variety of popular models, varying from linear discriminant analysis and the related ridge regression scheme to neural networks and support vector machines, all trainable on the data distribution. In order to disassociate feature selection from subsequent classification, we require the maximum performance of classification on the independent test-set. Thus, each gene-selection scheme is combined with all classifiers, optimized and tested through the first scenario on the independent test-set. The algorithmic parameters are optimized as to maximize performance on the independent test-set scenario, under the additional condition of correct classification of the training set. The classifier that achieves the best performance under this test is selected and used with its fixed parameters in all other evaluation tests. The algorithmic parameters are fixed based on the more difficult data set of breast cancer and are similarly used on all three data sets. This scenario is close to the operation of a decision support system for the categorization of new cases, where the classifier is used with fixed, already optimized parameters. Furthermore, when comparing algorithmic performance on different data sets, it is important to preserve the same algorithmic parameters in order to evaluate the stability of algorithms under a cross-experiment evaluation scheme. Table 12 summarizes the parameter values used for each approach in the conducted experiments for either gene selection or classification.

**Table 12:** Parameters used for all methods employed; parameters were selected in a way such that best performance was achieved on the test-set.

**Method Name**	**Weight Assignment Classifier**	**Accuracy Measure Classifier**	**Parameters Used for feature selection**	**Parameters Used for Classification**
RFE-SVM	SVM	SVM	C = 100	LK^†^, C = 100
RFE-LNW-GD	LNW-GD	SVM	*μ *= 10^-3^, Epochs = 500	LK^†^, C = 100
RFE-LSSVM	LSSVM	LSSVM	*γ *= 0.1	LK, *γ *= 0.1
RFE-RR	RR	SVM	a = 10^-1^	LK^†^, C = 1
RFE-FLDA	FLDA	SVM		LK^†^, C = 100

RFE-LNW1	LNW	SVM	*μ *= 10^-2 ^Epochs*	LK^†^, C = 100
RFE-LNW2	LNW	SVM	*μ *= 10^-4 ^Epochs^@^	LK^†^, C = 100
RFE-FSVs-7DK	FSV	SVM	7DK^‡^, C = 100	LK^†^, C = 100

Filter	Fisher's Ratio	SVM	-	LK^†^, C = 100

* Use 3000 epochs as long as the number of surviving genes is larger than 100 and a variant learning rate afterwards [26].

†Linear Kernel, ‡7 degree polynomial kernel

@ Use 300 epochs as long as the number of surviving genes is larger than or equal to 1024 and 500 epochs afterwards.

### Definition and Justification of Measures

Let *ν*_*i *_denote the measurement vector of the *i*^th ^individual, or the *i*^th ^sample of the data set, with *y*_*i *_denoting its associated class label. A classifier *C *trained on a given data set *D *maps an unlabelled instance *ν *∈ *V *to a label *y *∈ *Y*. The notation *C*(*D*, *ν*) indicates the label assigned to an unlabelled instance *ν *∈ *V *by a classifier built on data set *D*. In k-fold CV, the data set *D *is randomly divided into *k *mutually exclusive subsets (folds) *D*_1_,..., *D*_*k *_of approximately equal size. The classifier is trained and tested *k *times; each time *t *(*t *= 1,..., *k*) is trained on *D*\*D*_*t *_= {*D*-*D*_*t*_} and tested on *D*_*t*_. The "*performance profile" *is the matrix *S *of *m *rows and *n *columns, where *m *is the number of runs (folds) and *n *the number of subjects. We define each element *S*_*ij *_as follows:

(1) $$S_{ij}=\{\begin{array}{ll} \delta \left(C\left(D\backslash D_{k},v_{j}\right),y_{j}\right), & \forall \langle v_{j},y_{j}\rangle \in D_{k},i=1\ldots K\\ NULL, & otherwise \end{array}$$

where *K *corresponds to the total number of runs induced by the CV scenario and *δ*(.,.) indicates the Dirac delta function.

Along each row of matrix *S *we define the cardinality *C*_*Ri *_of row *i *and the cardinality *C*_*Pj *_of column *j *as the number of active entries (one or zero) within each row and column, respectively. Along each row of *S *we define the mean accuracy per CV run:

(2) $$acc_{R_{i}}=\frac{1}{C_{R_{i}}}\sum_{j=1}^{n}S_{ij}$$

which assess the model's generalization on the test-set, while keeping the training set fixed. We can also derive the confidence interval $CI_{R_{i}}$ for the classification of the testing set *D*_*i *_in this run based on a completely separate training set. Each run *R*_*i *_is a split-sample process [16], where the prediction outcomes for the samples in the testing set are truly independent Bernoulli trials, so that the derivation of a binomial confidence interval $CI_{R_{i}}$ is fully justified. Based on multiple split-sample runs, Michiels et al. [18] proposed a strategy for the estimation of CIs on the true prediction power of a method, by means of a percentile on the empirical distribution of multiple run estimates. In this study, we follow a similar approach for estimating measure of overall accuracy, considering the percentile distribution of accuracies either per run or per-subject. We employ the sample mean and standard deviation of per run accuracies in (3) to model multiple run estimates and derive measures for the mean prediction accuracy and its CI over all runs, denoted by the pair (*acc*_*R*_, *std*_*R*_). Notice that the accuracy measure *acc*_*Ri *_for the *i*^th ^run indicates how well the specific training-test can represent other (new) cases or samples (used in the testing-set). Consistently high accuracy for all CV runs (with different training-sets) indicates good overall prediction power and generalization ability of the method. Thus, the pair (*acc*_*R*_, *std*_*R*_) is employed as an index of the algorithmic performance and stability in its learning and generalization process. The 95% confidence interval (*CI*_*R*_) is set to 1.96 times the standard deviation *std*_*R*_.

We now turn our attention to measures along the columns of the performance profile *S *that reveal different issues of algorithmic performance. In this form, the mean per-subject accuracy is given by:

(3) $$acc_{P_{j}}=\frac{1}{C_{P_{j}}}\sum_{i=1}^{m}S_{ij}$$

We can also derive the confidence interval *CI*_*pj *_for the classification over all runs of subject *P*_*j*_. Nevertheless, the assumption of truly independent Bernoulli trials in the computation of CI breaks down, due to some overlap in the training sets across the runs [18]. Having denoted the potential of some bias in the computation of *CI*_*pj*_, we could use it with the due caution [16]. We also emphasize that such accuracy measures for individuals are highly dependent on the testing strategy, e.g. the design of the CV splits, which affects the number of iterations that this individual is tested for. Individuals that are randomly selected in the testing subset many times may yield more trustworthy measures on the per-subject performance of the algorithm, as well as tighter bounds on the CIs of accuracy. Individuals that are tested only a few times result in wider CIs, implying that they may influence the prediction of the overall algorithmic performance either in a positive or negative trend due to the small set random correlation effect. Such samples should be removed from the process of algorithmic evaluation. In our study we exclude subjects (samples) that have been tested less than ten times within the CV procedure. Similarly, per-subject measures may also be used to identify and clean up the data from irrelevant samples or samples highly affected by measurement noise.

The accuracy measure for each subject indicates how well this tested sample fits the model developed by the training process. In essence, high accuracy achieved for all CV runs indicates that the specific sample fits well to the decision space defined by all other samples. Consistently high accuracy of the algorithm for all tested samples indicates good and stable prediction characteristics. Thus, the mean accuracy *acc*_*p *_over all tested samples/subjects may be used as an index of the prediction power of an algorithm, similar to the overall accuracy per runs. The standard deviation *std*_*p *_of the per-subject accuracies over all tested samples is a measure of algorithmic stability on perturbations of the training and/or testing sets. For that reason, small standard deviation of per-subject accuracies of an algorithm indicates robustness to changes of the sample distributions. As in the previous case, the 95% confidence interval (*CI*_*P*_) is set to 1.96 times the standard deviation *std*_*P*_.

In order to exploit and compare performance statistics on various testing sets, we compute separately the average performances per run involving (i) the CV testing subjects, (ii) the samples in the independent test-set and (iii) the total set of tested samples.

### Consistency index

We use consistency index as a measure to estimate the stability of algorithms on the gene-selection process. This measure is based on gene frequencies over the CV iterations, as defined below:

(4) $$Concistency\;Index=\frac{\sum_{i=1}^{S}\left[F_{i}\right]}{S}$$

(5) $$F_{i}=\frac{O_{i}}{R},$$

where [*F*] defines a descent-ordered list of gene frequencies and *F*_*i *_is the frequency rate of gene *i *defined as its number of occurrences (*O*_*i*_) over the total number of runs (*R*). Furthermore, *S *is the total number of genes in the signature of each algorithm, defined on the basis of best performance on the independent test-set (Tables 1, 2, 3).

## Abbreviations

BC: Breast Cancer; CC: Colon Cancer; CI: Confidence Interval; CV: Cross Validation; RFE-FLDA: Recursive Feature Elimination based on Fisher's Linear Discriminant Analysis; RFE-FSVs-7DK: Recursive Feature Elimination based on Fisher's ratio of Support Vectors using a 7 Degree polynomial Kernel; RFE-LNW1, RFE-LNW2: Recursive Feature Elimination based on Linear Neuron Weights; RFE-LNW-GD: Recursive Feature Elimination based on Linear Neuron Weights using Gradient Descent; RFE-LSSVM: Recursive Feature Elimination based on Least Square Support Vector Machines; RFE-RR: Recursive Feature Elimination based on Ridge Regression; RFE-SVM: Recursive Feature Elimination based on Support Vector Machines.

## Authors' contributions

MZ and MEB contributed equally to the work. MZ drafted most of the manuscript, designed the evaluation on a per run and a per-subject basis and performed data analysis on this part. MEB run the experiments, wrote all MatLab codes and contributed to data analysis for the independent test-set and 10-fold cross validation scenarios, while he assisted in drafting the manuscript. DK, VD, GT and MT assisted with the study design, data analysis and drafting of the manuscript. GT assisted with the statistical analysis of results. All authors read and approved the final manuscript.
